# The Future Role of Radiologists in the Artificial Intelligence-Driven Hospital

**DOI:** 10.1007/s10439-024-03556-3

**Published:** 2024-06-07

**Authors:** Sam Sedaghat

**Affiliations:** Department of Diagnostic and Interventional Radiology, University Hospital of Heidelberg, Im Neuenheimer Feld 400, 69120 Heidelberg, Germany

**Keywords:** Artificial intelligence, ChatGPT, Hospital, Radiology, Imaging, Medicine

## Abstract

Increasing population and healthcare costs make changes in the healthcare system necessary. This article deals with ChatGPT’s perspective on the future role of radiologists in the AI-driven hospital. This perspective will be augmented by further considerations by the author. AI-based imaging technologies and chatbots like ChatGPT can help improve radiologists’ performance and workflow in the future AI-driven hospital. Although basic radiological examinations could be delivered without needing a radiologist, sophisticated imaging procedures will still need the expert opinion of a radiologist.

## Introduction

Increasing population and healthcare costs make changes necessary, which could be accelerated by artificial intelligence (AI), helping to make hospitals more efficient and allowing more precise healthcare delivery. Radiology is the frontrunner in AI in medicine, and many imaging-based AI applications have already been developed for patient-centred medicine. Examples of AI-based imaging applications include applications for prevention and diagnostics of cardiovascular diseases [1] or automatic analysis of spine fractures [2]. Another AI-based application is ChatGPT, which was introduced at the end of 2022 and soon after attracted millions of people. ChatGPT is an AI chatbot that has the potential to change healthcare professionals’ lives in many ways, such as by mitigating the way medical resources are extracted [3–6]. In this article, ChatGPT’s perspective on the future role of radiologists in the AI-driven hospital is investigated. ChatGPT’s perspective will be augmented by further considerations by the author.

## What is the Role of Radiologists in the Future AI-Driven Hospital According to ChatGPT?

1. Enhanced diagnostic accuracy, especially in complex cases [7].
2. Efficiency and workflow improvements, thereby allowing radiologists to focus more on complex cases [7].
3. Developing advanced imaging techniques, especially in offering new ways to visualize and interpret medical images [7].
4. Improved training and education by offering simulated cases or providing feedback on diagnostic decisions, for example [7].
5. Improved personalized medicine by integrating data into clinical history or other diagnostic data, for example [7].
6. Expanded role in patient care by involving radiologists more in counselling patients about imaging findings [7].
7. Enhanced research and development, especially in the refinement of AI technologies [7].

## What is the Synergetic Effect of ChatGPT and Radiologists in the Future AI-Driven Hospital According to ChatGPT?

1. Enhanced diagnostic support by helping radiologists process and synthesize vast amounts of clinical data to support diagnostic decisions [7].
2. Improved efficiency by managing routine tasks such as drafting radiology report templates or summarizing patient histories, for example [7].
3. Enhanced patient communication and education by providing understandable explanations of imaging findings, answering common questions about procedures or clarifying follow-up steps [7].
4. Enhanced training and continuing education by simulating interactive case studies, offering quizzes, or providing up-to-date medical content [7].
5. Research assistance by helping with literature reviews, data analysis, and drafting of research papers or grant applications [7].
6. Clinical decision support by providing a more comprehensive decision-support system, offering insights from a broader range of data sources [7].
7. Facilitating interdisciplinary collaboration by helping to summarize patient data and imaging interpretations [7].

## Further Considerations

AI-based imaging and chatbots like ChatGPT could augment medical practice in many ways in the future. To date, radiologists are routinely consulted when diagnostics come to radiological imaging, ranging from simple X-rays to sophisticated MRI examinations. This picture of “today” could tremendously change by “tomorrow” due to the emergence of AI. In the future, in AI-augmented medical departments, basic diagnostic examinations could be automatically analysed, and results presented to physicians without the need to consult a radiologist. This could relieve radiologists by reducing their workload and reducing the time to the availability of diagnostic results. In the emergency setting, for example, where time to diagnostic results influences patients’ outcomes, timely radiological results and findings are essential. To draw the bigger picture, radiological processes within the medical department (e.g. emergency department) could become partially or fully automated, eventually leading to more efficient delivery of diagnostic results. This might start from the admission to the department, where chatbots could evaluate the need for radiological diagnostics during the automated evaluation of patients’ history and might end in the mentioned automatic delivery of radiological procedures, consecutively leading to novel and standardized healthcare delivery pipelines and optimized imaging procedures. However, sophisticated imaging procedures and examinations will still need the expert opinion of a radiologist. In these cases, AI applications could help radiologists evaluate imaging findings more efficiently, eventually assisting radiologists in dealing with the increasingly high volume of images. One might assume that radiologists will be obsolete one day by the emergence of AI. That assumption might be valid for easy-to-handle imaging procedures but not for sophisticated radiological examinations. Consecutively, the consultation of radiologists will be shifted to a more sophisticated level of examinations with the emergence of AI. However, we are still at the beginning of this technological revolution, and the future will generate many more highly sophisticated imaging-based AI applications for use in medicine.

## Conclusion

AI-based imaging technologies are essential for the future of medicine. AI will spawn many advantages and chances for radiological services in medicine, such as enhancing diagnostic accuracy or improving efficiency and workflow. Chatbots like ChatGPT can help improve radiologists’ performance and workflow in the future AI-driven hospital. However, in the future AI-driven hospital, basic radiological examinations could be automated and delivered without needing a radiologist. However, sophisticated imaging procedures and examinations will still need the expert opinion of a radiologist.
